# Synergistic Effect of Doxorubicin and Blue Light Irradiation on the Antitumor Treatment of HepG2 Cells in Liver Cancer

**DOI:** 10.3390/molecules29143360

**Published:** 2024-07-17

**Authors:** Yun Teng, Zhige Li, Junsong Liu, Lesheng Teng, Hongdong Li

**Affiliations:** 1 State Key Laboratory of Superhard Materials, College of Physics, Jilin University, Changchun 130012, China; tengyun18@mails.jlu.edu.cn; 2 School of Life Sciences, Jilin University, Changchun 130012, China; lizg21@mails.jlu.edu.cn

**Keywords:** HepG2 cell, blue light irradiation, DOX, proliferation inhibition, apoptosis

## Abstract

Doxorubicin (DOX) has been an effective antitumor agent for human liver cancer cells; however, an overdose might lead to major side effects appearing in clinical applications. In this work, we present a strategy of combining DOX and blue light (BL) irradiation for the antitumor treatment of HepG2 cells (one typical human liver cancer cell line). It is demonstrated that synergetic DOX and BL can significantly reduce cell proliferation and increase the apoptotic rate of HepG2 cells in comparison to individual DOX treatment. The additional BL irradiation is further helpful for enhancing the inhibition of cell migration and invasion. Analyses of reactive oxygen species (ROS) level and Western blotting reveal that the strategy results in more ROS accumulation, mitochondrial damage, and the upregulation of proapoptotic protein (Bcl-2) and downregulation of antiapoptotic protein (Bax). In addition to the improved therapeutic effect, the non-contact BL irradiation is greatly helpful for reducing the dosage of DOX, and subsequently reduces the side effects caused by the DOX drug. These findings offer a novel perspective for the therapeutic approach toward liver cancer with high efficiency and reduced side effects.

## 1. Introduction

Liver cancer is a prevalent worldwide malignancy with an escalating incidence [1,2]. It ranks as the sixth most common cancer globally and represents the third leading cause of cancer-related mortality [1]. Hepatocellular carcinoma (HCC) is the most common form of liver cancer, accounting for about 90.1% of cases [3]. Various methods have been proposed for the treatment of HCC, including surgical resection, liver transplantation, radiation therapy, percutaneous ablation techniques, locoregional therapies and systemic pharmacotherapies [4,5,6,7]. Nevertheless, the five-year survival rate of HCC remains as low as 14.1% [8], and enhancing therapeutic efficacy and achieving higher cure rates are a challenge thus far. Being a potent cytotoxic chemotherapy drug commonly employed for the treatment of solid tumors [9,10,11] and hematological malignancies [12,13], doxorubicin (DOX) has been served as an important therapeutic agent in the clinical management of the liver cancer [14,15]. However, high doses of DOX might cause cardiotoxicity-induced congestive heart failure [16,17]. Normally, DOX is administered in combination with other chemotherapeutic agents, including cisplatin [18], paclitaxel [19], and 5-fluorouracil [20], to increase its efficiency. Therefore, ensuring the therapeutic effectiveness of DOX with fewer side effects is desirable for the treatment of liver cancer. DOX’s inherent photosensitive property has been applied in combination with photodynamic therapy to enhance its anticancer activity against tumor cells [21,22].

Blue light (BL) irradiation has been systematically investigated in tumor treatments related to apoptosis, proliferation inhibition, autophagy, migration inhibition, etc. [23,24,25,26,27]. For example, BL irradiation was proposed to induce apoptosis in the human promyelocytic leukemia cell line of HL60 and Kasumi cells by the mitochondrial pathway [26,27]. Under BL irradiation, the apoptosis of colon cancer was enhanced with increasing ROS production stimulating autophagy [28,29]. Especially, combining BL irradiation and arsenic trioxide (ATO) has significantly enhanced the therapeutic efficacy on osteosarcoma cells at related low ATO doses [30]. Exposure to BL irradiation could enhance the sensitivity of bladder cancer cells towards cisplatin treatment, which is helpful for further reducing the drug doses and the corresponding side effects of chemotherapy [31]. Compared with DOX alone, combing low-power BL irradiation and DOX resulted in enhancing cytotoxicity to MDA-MB-231 cultures and increasing ROS levels [22].

Recently, our group demonstrated that under sole BL irradiation, the common human liver cancer cell lines of SMMC-7721 and HepG2 exhibited a certain extent of proliferation inhibition and mitochondrial damage, leading to promoting apoptosis of the cells [32]. Taking into account the synergistic effect of traditional drug treatment and photodynamic therapy, it is speculated that combining DOX and BL irradiation is a useful strategy to realize the highly efficient treatment of liver tumors. In this work, the HepG2 cell line (one of the most common types of HCC cell lines of liver cancer [33]) is selected for examination. The proliferation inhibition, apoptosis-related protein regulation, and migration and invasion of HepG2 cells are experimentally investigated to reveal the effects of sole BL, DOX, and combined BL/DOX therapies. It is demonstrated that synergic BL irradiation and DOX shows a better therapeutic effect with respect to the cases of sole BL or DOX, and the improvements are mainly attributed to a BL-induced increase in ROS accumulation, mitochondrial damage, and cysteine protease activation.

## 2. Results and Discussion

**Figure 1:**
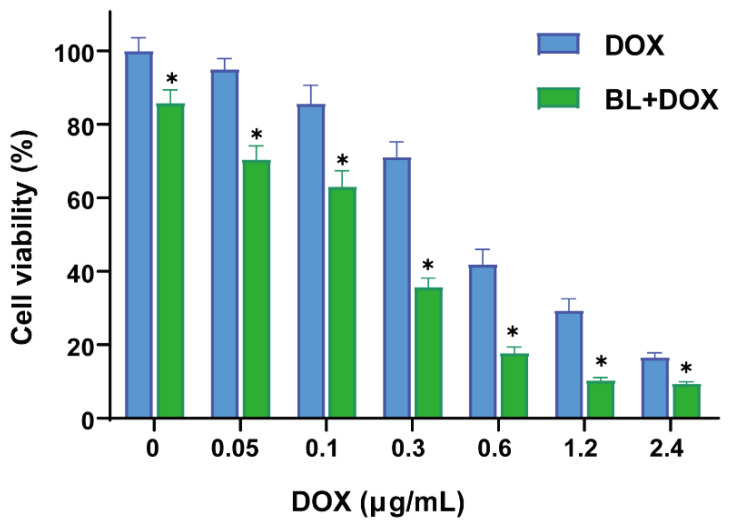
Cell viability rates of HepG2 cells treated for 24 h by DOX alone and combined BL irradiation and DOX at varying concentrations. *n* = 6; * *p* < 0.001.

For the DOX treatment of HepG2 cells, HepG2 cells were incubated in 100 μL DMEM solution with various DOX concentrations of 0, 0.05, 0.1, 0.3, 0.6, 1.2 and 2.4 in μg/mL. In Figure 1, the results of the CCK-8 assay reveal that the number of dead cells of HepG2 monotonously increases at higher DOX doses. The proliferation inhibition of HepG2 cells is DOX concentration dependent, and the DOX IC_50_ value is about 0.45 μg/mL (Figure S1). In addition, the cell viability rates of HepG2 cells under BL irradiation at 438 nm (Figure S2) for 10 min, 20 min, 40 min, and 60 min are 95.07%, 64.48%, 32.39%, and 18.07%, respectively (Figure S3). Combining 10 min BL irradiation and DOX, a superior therapeutic effect is presented for the proliferation inhibition of HepG2 cells at the corresponding concentrations of DOX. The optimized experimental condition for the following study is thus BL irradiation for 10 min and additive DOX at 0.3 μg/mL.

**Figure 2:**
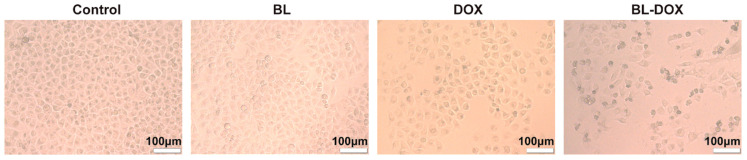
Representative optical photographic images of HepG2 cells treated under different conditions of the control, BL irradiation, DOX, and combined BL-DOX (from **left** to **right**). Scale bar: 100 μm.

The morphological features of the HepG2 cells after treatments under different conditions are presented in Figure 2. In comparison to the control groups, treated with sole 10 min BL irradiation or 0.3 μg/mL DOX, the density of the adherent cells is decreased, and after treatment by combined BL/DOX, more floating dead cells appear in the sample. As shown in Figure S4, the contracted rate of HepG2 cells for the treatment of BL/DOX is 5.1 (2.8) times that for the treatment of BL irradiation (DOX), indicating the strong synergistic effect of combining BL and DOX, enhancing the killing efficacy for HepG2 cells. This morphology evolution of the treated cells further confirms that the synergistic effect of combined BL and DOX enhances the killing efficacy for HepG2 cells. We performed the cell viability test of a typical normal liver cell of LO2 (Figure S5 in Supplementary Materials). After BL irradiation for 20 min, the viability rate of LO2 cells maintains 92.5% (*p* > 0.05), implying that normal cells are safe under BL irradiation.

**Figure 3:**
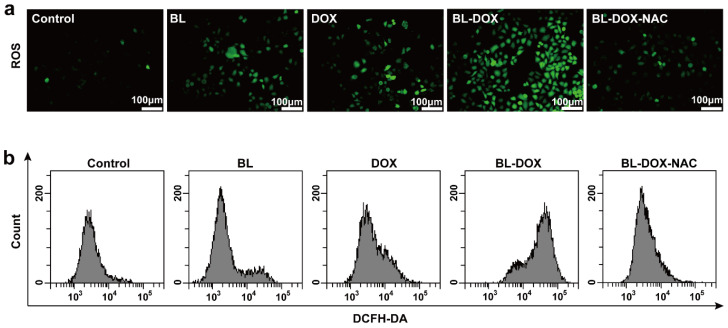
(**a**) Intracellular levels of ROS production in HepG2 cells before and after treatments of BL, DOX, BL-DOX, and BL-DOX-NAC. (**b**) ROS fluorescence intensity detected by flow cytometry. *n* = 3.

Normally, an elevated ROS level in cells can cause oxidative damage of cellular molecules (e.g., lipids, proteins, and DNA), resulting in the occurrence of apoptosis [34,35]. To interpret the mechanism of the suppression of HepG2 cells enhanced by combining the drug and BL irradiation, the DCFH-DA staining examinations related to ROS were carried out. As shown in Figure 3, the synergistic DOX and BL irradiation treatment can significantly increase the ROS level with respect to the case of either sole DOX or BL irradiation. In addition, it is confirmed that the greater ROS generation is indeed related to introducing BL and/or DOX, as ROS has been significantly inhibited by adding ROS scavengers (N-acetyl cysteine (NAC)) in the culture solution.

**Figure 4:**
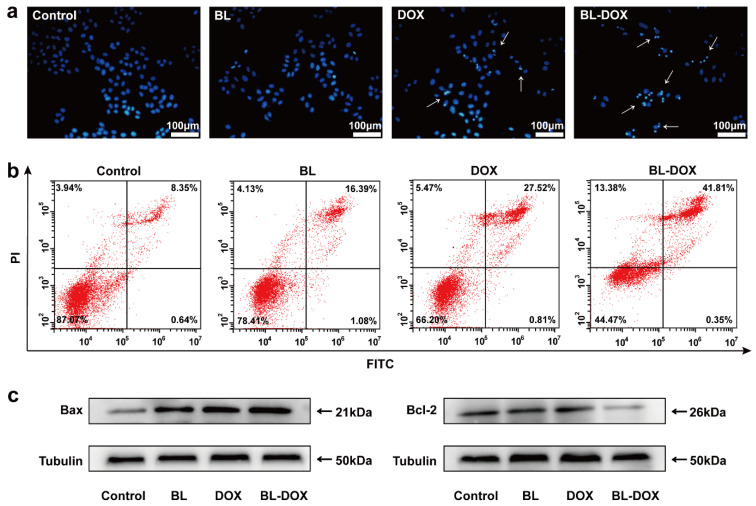
(**a**) Fluorescent staining photograph of HepG2 cells before and after treatment with BL, DOX, and BL-DOX. The arrows indicate apoptotic cells. Scale bar: 100 μm. (**b**) Annexin V-FITC/PI apoptosis detection assay, flow cytometry analysis indicating the total apoptosis ratios of apparent early apoptosis (lower right quadrant) and late apoptosis (upper right quadrant). The red dots represent cells. (**c**) Expressions of Bax and Bcl-2 proteins in HepG2 cells by Western blot. *n* = 3.

The apoptosis is generally related to the ROS-enhanced release of mitochondrial contents by decreasing (increasing) mitochondrial membrane potential (mitochondrial outer membrane permeability (MOMP)) [36]. It is thus reasonable that DOX combined with BL irradiation can enhance mitochondrial apoptosis compared to the drug or BL irradiation alone due to more ROS being excited. A previous report demonstrated that DOX induces oxidative stress caused by generating ROS in HepG2 cells to trigger apoptosis [37]. The impact of the combination therapy of DOX and BL irradiation on HepG2 cell apoptosis was assessed through nuclear staining and flow cytometry analysis, as observed by blue fluorescence from the apoptotic cell nuclei (Figure 4). In Figure 4a, the nuclei in the control group have a nearly identical shape and size, well-defined boundaries, and uniform staining. After being treated by BL irradiation or DOX, a few of the nuclei are decreased in size and show enhanced fluorescence. Importantly, in the BL/DOX group, both the number and size of the HepG2 cell nuclei are significantly decreased, and the chromatin condensation and blue fluorescence enhancement are evidently presented, which are all attributed to the occurrence of improving apoptosis. In Figure 4b, the flow cytometry results reveal an increased percentage of apoptotic cells in HepG2 cell lines following DOX or BL irradiation treatment. The apoptotic cell population rises from approximately 8.99% of the control to 17.47% with BL irradiation and 28.33% with DOX monotherapy. Notably, treated by the combined DOX and BL irradiation, the apoptotic rate significantly increases up to 42.16%. Upon the occurrence of apoptosis, the nuclear morphology of HepG2 cells (assessed by Hoechst staining) undergoes changes and shrinks to form a condensed nucleus. These data further confirm the synergistic enhancement of antitumor effects on HepG2 cells by the combined treatment of DOX and BL irradiation.

The potential impacts of DOX and BL irradiation on the modulation of the apoptosis and proliferation factors of HepG2 cells are further verified by Western blotting. Normally, the Bcl-2 family (i.e., Bax, Bad, Bcl-2 and Bcl-xl, etc.) plays a crucial role in determining the process of apoptosis [38,39]. Specifically, the expression levels of Bax and Bcl-2 in HepG2 cells were systematically examined, which are involved in the apoptotic pathway. The increase in proapoptotic protein and decrease in antiapoptotic protein indicate the initiation of the apoptosis process. In Figure 4c, the general trend is that the expression levels of Bax, (Bcl-2) are increased (decreased) after treatments of BL irradiation, DOX, and BL-DOX in comparison to the control. In addition, for the three treatment processes, the strength in increment (decrement) of those proteins follows the order of BL-DOX > DOX > BL (BL > DOX > BL-DOX). The combination therapy of BL-DOX can suppress the Bcl-2 pathway, induce ROS production, and inhibit DNA synthesis to promote apoptosis in HepG2 cells. The expression levels of these aforementioned proteins support apoptosis in HepG2 cells treated by BL, DOX, and combined BL-DOX.

**Figure 5:**
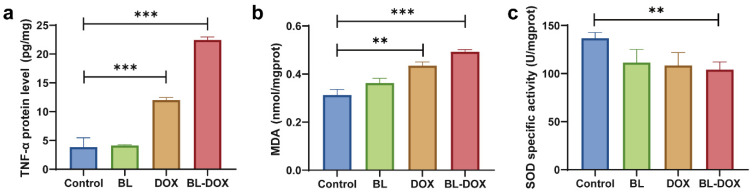
(**a**) TNF-α protein level, (**b**) alteration of MDA, and (**c**) SOD activity for HepG2 cells with BL, DOX, and combined BL-DOX treatments. *n* = 3; ** *p* < 0.01, *** *p* < 0.001.

As is known, tumor necrosis factor-α (TNF-α) is a crucial inflammatory cytokine which can trigger the exogenous caspase-8 activation pathway and induce cell apoptosis by binding caspase-8 to Fas-associating protein with a novel death domain (FADD) [40]. These signals mainly transduce to mitochondria, and then a series of biochemical events occur in the mitochondria, leading to the MOMP [40,41]. The elevated intracellular ROS level results in generating oxidative byproducts such as malondialdehyde (MDA), which is commonly measured to assess the extent of oxidative stress [42,43]. The superoxide dismutase (SOD) plays a vital role in preventing cellular oxidative damage by scavenging ROS, and in addition, SOD can also regulate the metabolic process of cells and reduce the production of ROS to further enhance the antioxidant capacity of cells [44]. In Figure 5, in comparison with the control group, both DOX and BL/DOX groups have significant increases in TNF-α protein and MDA levels, and in contrast, the total SOD enzyme activity is decreased. These results strongly support the fact that for the antitumor treatment of HepG2 cells, the synergetic effect of combined BL radiation and DOX (i.e., phototherapy with chemotherapy) is highly effective by enhancing oxidative stress and apoptosis.

**Figure 6:**
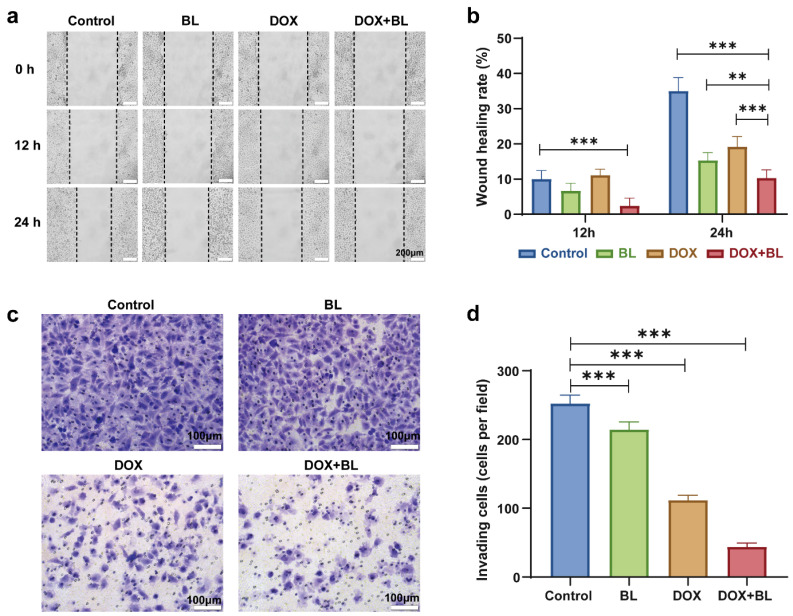
Cell migration and invasion assay for the HepG2 cells with BL, DOX, and combined BL-DOX treatments. Representative optical photograph images (**a**), corresponding statistics histogram of calculated percentages (**b**) for wound-healing assay at 12 h and 24 h after treatment. Crystal violet staining images (**c**) and invasion cells statistics histogram (**d**) for 24 h transwell assay. Scale bars: 200 μm for (**a**), 100 μm for (**c**). *n* = 5; ** *p* < 0.01, *** *p* < 0.001.

To further assess the synergetic effect of BL irradiation and DOX on the treatment of HepG2 cells, the corresponding migration and invasion of the cells were tested by wound healing and transwell assays. Obviously, as seen in Figure 6a,b, the combination treatment of BL and DOX significantly suppresses the migration of HepG2 cells in comparison to treatments with BL irradiation, DOX, or the control group. Furthermore, the invasion experimental results of HepG2 cells (Figure 6c,d) indicate that the suppressive effect is enhanced following the combination treatment of BL plus DOX with respect to the cases of sole BL or DOX. Epithelial–mesenchymal transformation (EMT) is generally regarded as an important biological process that ultimately leads to cancer invasion and metastasis [45]. The EMT process might be a mechanism for the migration and invasion of HepG2 cells, which is similar to the case of inhibiting the EMT process in colorectal cancer cells under BL irradiation [28,29].

## 3. Materials and Methods

### 3.1. Materials

The HepG2 cells were sourced from the Cell Bank of the Shanghai Academy of Chinese Sciences (SCSP-510, Shanghai, China). The LO2 cells were sourced from Cellverse Bioscience Technology Co., Ltd. (iCell-h054, Shanghai, China). Dulbecco’s modified eagle medium (DMEM) was acquired from Thermo Fisher Scientific (Shanghai, China). Doxorubicin hydrochloride (DOX•HCl, 98%) was acquired from Aladdin Reagent Co., Ltd. (Shanghai, China). The ROS Assay Kit, NAC, Cell Counting Kit-8 (CCK-8), Annexin V-FITC/PI Apoptosis Kit, 0.1% crystal violet, and Hoechst 33258 were sourced from Beyotime Biotechnology (Shanghai, China). The Human TNF-α ELISA Kit (E-EL-M3063), Malondialdehyde (MDA) Colorimetric assay kit (E-BC-K028-M), and Total Superoxde Dismutase (T-SOD) Activity Assay Kit (E-BC-K020-M) were acquired from Elabsecience (Wuhan, China). The BCA Protein Assay Kit was acquired from Thermo (Shanghai, China), and transwell filter chambers were sourced from Corning Incorporated (New York, NY, USA). All the antibody information is shown in Table S1.

### 3.2. Cell Culture

HepG2 cells and LO2 cells were cultured in DMEM containing 4500 mg/L glucose, 10% FBS, and 1% penicillin-streptomycin. All the cells were maintained at 37 °C in a humidified atmosphere containing 5% carbon dioxide.

### 3.3. BL Irradiation System for Cell Treatments

The irradiation panel (size: 15 × 12 cm^2^) system consisted of 192 BL LED beads with a wavelength centered at 438 nm (Figure S2) and a power density of 60 mW/cm^2^ (tested by an optical power meter CEL-NP2000). The cell culture flask was placed above the LED panel, and the distance between the light source and cells in the culture box was 4 cm. In the experiments of the combined treatment of BL irradiation and DOX, the HepG2 cells were first exposed under BL irradiation for 10 min (energy density of 36 J/cm^2^) and subsequently cultured with DOX at varying doses for 24 h.

### 3.4. Growth Viability Assay

The cells were seeded onto 96-well plates at a density of 1 × 10^4^ cells per well and cultured for 24 h. Subsequently, each well was treated with an addition of CCK-8 solution (10 μL) and incubated for an additional period of 2 h at 37 °C. The optical density (OD) was examined at wavelength λ = 450 nm measured by a Spectra Max^®^ Absorbance Reader to evaluate cell growth inhibition.

### 3.5. ROS Examination

ROS levels were measured by an ROS Assay Kit. The cells were seeded at a density of 1 × 10^5^ per well in 6-well plates. With BL irradiation and/or DOX treat for 4 h, the cells were washed twice with PBS after the removal of media and incubated with 10 mM DCFH-DA (1:1000 dilution) probes for 30 min at 37 °C. The cell images were tested by a fluorescence microscope (OLYPUS BX51, Olypus Corporation, Tokyo, Japan) and flow cytometry (CytoFLFX, Beckman, New York, NY, USA). To verify ROS production and the promotion of cell apoptosis by the BL-DOX treatment, an ROS scavenger of N-acetyl cysteine (NAC) was added in the culture solution for comparison.

### 3.6. Apoptosis Analysis

Apoptosis of the cells was assessed using the Annexin V-FITC/PI reagent. With 1 × 10^5^ per well, all cells were seeded into 6-well plates and incubated for 24 h. After treatment, the collected cells were resuspended in a 200 μL assay buffer, then 10 μL Annexin V-FITC and 5 μL PI reagents were added following an incubation for 20 min at 37 °C. All samples were analyzed by flow cytometry.

### 3.7. Intracellular TNF-α Assay

TNF-α activity was tested according to the instructions of the enzyme-linked immunosorbent assay (ELISA) kit. Briefly, HepG2 cells were seeded in 6-well plates at a density of 2 × 10^5^ cells/well for 24 h, and then treated with DOX and/or BL for another 24 h. TNF-α activity was measured according to the manufacturer’s protocol, and the data reading was taken at 450 nm using a Spectra Max^®^ Absorbance Reader (HBS-1096A v3.0).

### 3.8. Detection of Superoxide Dismutase (SOD) Activity

SOD activity was tested according to the instructions of the commercial kit as described. HepG2 cells were seeded in 6-well plates at a density of 2 × 10^5^ cells/well for 24 h and then treated with DOX and/or BL for another 24 h. Total protein samples were collected, and the SOD mixture was incubated at 37 °C for 20 min. The absorbance was determined at 450 nm by spectrophotometry with a Spectra Max^®^ Absorbance Reader. One unit of SOD activity was defined as the amount of enzyme necessary to produce a 50% inhibition of the nitroblue tetrazolium reduction rate measured at 550 nm. SOD activity was expressed as SOD U/mg protein.

### 3.9. Malondialdehyde (MDA) Examination

MDA was measured by an MDA Colorimetric assay kit. HepG2 cells were seeded in 6-well plates at a density of 2 × 10^5^ cells/well for 24 h and then treated with DOX and/or BL for another 24 h. After being washed with PBS, the cells were homogenized and reacted with thiobarbituric acid (TBA) to form an MDA–TBA adduct. The absorbance of the MDA–TBA adduct was recorded at 532 nm by a Spectra Max^®^ Absorbance Reader.

### 3.10. Hoechst 33258 Staining

With 1 × 10^5^ per well, the HepG2 cells were seeded in 6-well plates. The cells were fixed with PBS, following incubation with 10 mM Hoechst 33258 (1:100 dilution) for 20 min at 25 °C. Fluorescence intensity was measured by a fluorescence microscope (20×).

### 3.11. Cell Migration Assay and Cell Invasion Assay

The scratch tests were performed to determine the cell migration in vitro. The cells were cultured in 6-well plates at a density of 2 × 10^5^ cells/well. A wound was created by scraping the cells with a 200 μL pipette tip on the confluent cell monolayer. After two times of washing with PBS, the cells were maintained in DMEM (0.4% FBS). Images were recorded for 0 h, 12 h, and 24 h treatment, respectively. The wound area was measured with ImageJ software (ImageJ 1.45, NIH, Bethesda, MA, USA).

Invasion assays were performed in transwell filter chambers, a 24-well tissue culture plate with inserts. The inserts contain an 8 μm pore size polycarbonate membrane. A total of 600 μL of DMEM medium containing 10% FBS was added to the lower chamber, and a cell suspension containing 7 × 10^4^ cells in 200 μL of serum-free DMEM medium was added to each insert and then incubated for 24 h. Invasive cells on the lower surface of the membrane were stained by dipping inserts in 4% paraformaldehyde for 5 min and 0.1% crystal violet for 15 min, following two times of the PBS washing process. Five visual fields of each insert were randomly chosen under a fluorescence microscope, and the invasion cell population was evaluated by ImageJ software.

### 3.12. Western Blot

With 2 × 10^5^ cells per well, the HepG2 cells were seeded in 6-well plates. After treatment, the total protein was extracted from the cells through a RIPA lysis buffer containing 1 mM PMSF. The protein quantification was conducted through a BCA Protein Assay Kit, and the concentration was determined by the BCA method. The total protein was separated by 10% SDS-PAGE. After complete separation by electrophoresis, the protein was transferred to polyvinylidene difluoride membranes by the semidry method. After incubation at 25 °C for 20 min with a rapid sealing solution, the membranes were incubated with a polyclonal rabbit antihuman dilution at 4 °C overnight. The membranes were then incubated with a horseradish peroxidase-conjugated secondary antibody for 4 h and washed six times with TBS. The immunoreactivity bands were visualized by Image Lab 4.0.1 software from Bio-Rad (Hercules, Shanghai, China) to calculate the relative expression of each protein.

### 3.13. Statistical Analysis

Statistical analysis was performed using GraphPad Prism (GraphPad Software 8.0.2). Data are presented as the mean ± SD. The significance of differences between the two groups was compared by an independent sample *t*-test, and the experimental results between multiple groups were compared by a one-way analysis of variance (one-way ANOVA). The value of *p* < 0.05 was considered statistically significant.

## 4. Conclusions

In this work, we propose a novel therapy strategy that enhances the proliferation inhibition and apoptosis of HepG2 cells, one typical line of HCC liver tumors, by BL radiation combined with DOX. The results demonstrate that this combined phototherapy and chemotherapy effectively promotes cell apoptosis and inhibits cell migration and invasion. BL irradiation increases the sensitivity of HepG2 cells to drugs, allowing for a reduced dosage of DOX and potentially minimizing the side effects associated with drug accumulation. Consequently, the combination of BL and DOX holds promise as a potential candidate for clinical tumor treatment, offering a synergistic approach alongside conventional drugs, photosensitizers, radiotherapy, and/or chemotherapy. Further animal model experiments are underway to verify the feasibility of the BL-DOX treatment on liver tumors.

## Data Availability

The data used to support the findings of this study are available from the corresponding author upon request.

## Supplementary Materials

The following supporting information can be downloaded at https://www.mdpi.com/article/10.3390/molecules29143360/s1, Figure S1: Variation of HepG2 cell viability rates treated by DOX; Figure S2: Emission spectrum of the blue LED beads; Figure S3: Variation of HepG2 cell viability rates treated by BL irradiation; Figure S4: Contracted HepG2 cell rates treated by BL irradiation, DOX, and BL-DOX; Figure S5: The safety effect of BL irradiation on normal liver LO2 cells; Table S1: Detailed information of antibodies used in western blotting.
