# Supplementary material for: Synergistic Effect of Doxorubicin and Blue Light Irradiation on the Antitumor Treatment of HepG2 Cells in Liver Cancer
Source: Molecules. 2024 Jul 17;29(14):3360. doi: 10.3390/molecules29143360 (PMC11279636; doi:10.3390/molecules29143360)
Supplement: Supplementary file 1 [file molecules-29-03360-s001.zip › molecules-3048429-supplementary.pdf]

Supplementary Information

Synergistic effect of doxorubicin and blue light irradiation on anti-tumor treatment of HepG2 cell

Yun Teng[a], Zhige Li[b], Junsong Liu[a]*, Lesheng Teng[b]*, Hongdong Li[a]*

[a]*State Key Laboratory of Superhard Materials, College of Physics, Jilin University, Changchun 130012, PR China*
[b]*School of Life Sciences, Jilin University, Changchun 130012, PR China*

***Correspondence authors**

Table S1. Detailed information of antibodies used in western blotting

Antibody	Molecular weight	Catalog number	Dilution for Western blot	Company*
Bax	21 kDa	A19664	1:2000	Abclonal
Bcl2	26 kDa	A02777	1:2000	Abclonal
GAPDH	50 kDa	Ab179513	1:2000	Abcam
Goat Anti-Rabbit (H+L)	/	E-AB-1003	1:2000	Elabscience
Goat-Anti-Mouse (H+L)	/	E-AB-1001	1:2000	Elabscience

*The all companies are in China.

Fig. S1 of 5

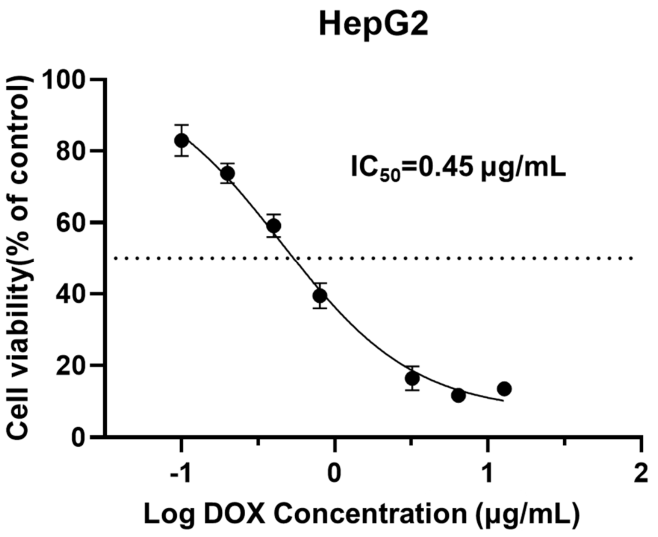

Fig. S1 Variation of HepG2 cell viability rates treated by DOX at concentrations of 0, 0.1, 0.2, 0.4, 0.8, 3.2, 6.4, and 12.8 μg/mL, in turn, tested in 10 μL CCK-8 solution. The half inhibitory concentration (IC_{50}) is defined as the DOX concentration with cell viability reduced to 50%, calculated by GraphPad Prism (GraphPad Software 8.0.2). *n*=6.

IC_{50} = Antilog [B+ (50-B)/(A–b)] × C, where A = log>50% drug concentration; B = log<50% drug concentration; C = log dilution.

Fig. S2 of 5

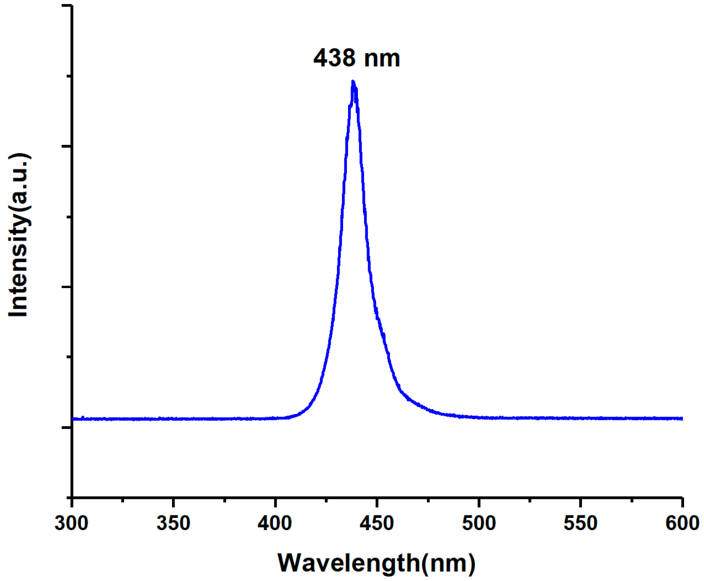

Fig. S2 Emission spectrum of the blue LED beads with a wavelength centered at 438 nm.

Fig. S3 of 5

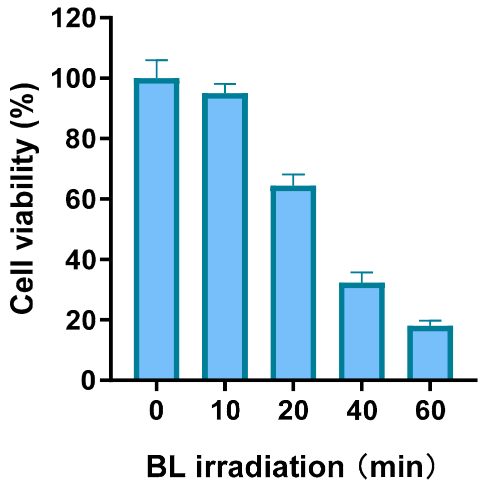

Fig. S3 Variation of HepG2 cell viability rates treated by BL irradiation for varying time. n=6.

Fig. S4 of 5

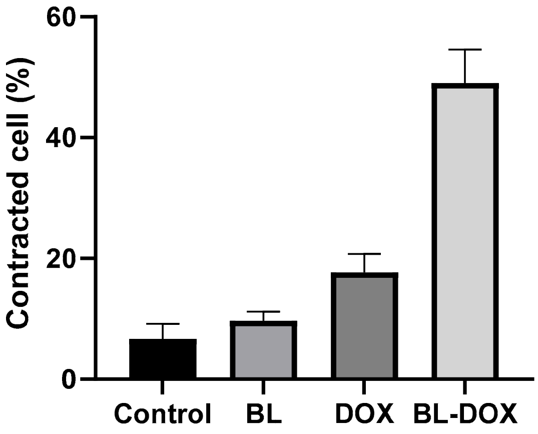

Fig. S4 Contracted HepG2 cell rates (calculated by ImageJ software) treated by BL irradiation, DOX, and BL-DOX. n=3

Fig. S5 of 5

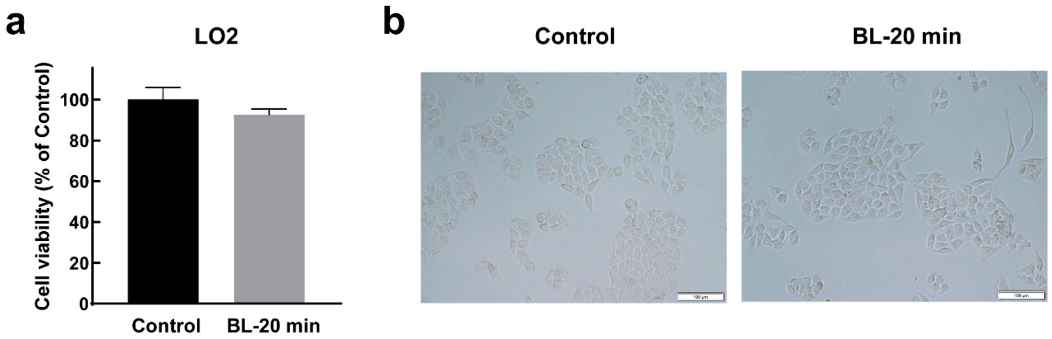

Fig. S5 The safety effect of BL irradiation on normal liver LO2 cells. **(a)** Variation of viability rate for normal liver LO2 cells treated by BL irradiation. **(b)** Representative optical photograph images of LO2 cells. *n*=6. Scale bar:100 μm.
